# Phylogenetic Inference of the 2022 Highly Pathogenic H7N3 Avian Influenza Outbreak in Northern Mexico

**DOI:** 10.3390/pathogens11111284

**Published:** 2022-11-01

**Authors:** Roberto Navarro-Lopez, Wanhong Xu, Ninnet Gomez-Romero, Lauro Velazquez-Salinas, Yohannes Berhane

**Affiliations:** 1 United States-Mexico Commission for the Prevention of Foot-and-Mouth Disease and Other Exotic Disease Animals, Mexico City 64590, Mexico; 2 National Centre for Foreign Animal Disease, Winnipeg, MB R3E 3M4, Canada; 3 Plum Island Animal Disease Center, Agriculture Research Service, USDA, Orient, NY 11944, USA; 4 Department of Animal Science, University of Manitoba, Winnipeg, MB R3T 2S2, Canada

**Keywords:** avian influenza virus, H7N3, highly pathogenic, phylogenetic analysis, transmission network, genetic diversity, antigenic evolution

## Abstract

The Mexican lineage H7N3 highly pathogenic avian influenza virus (HPAIV) has persisted in Mexican poultry since its first isolation in 2012. To date, the detection of this virus has gradually expanded from the initial one state to 18 states in Mexico. Despite the HPAIV H7N3 outbreak occurring yearly, the transmission pathways have never been studied, disallowing the establishment of effective control measures. We used a phylogenetic approach to unravel the transmission pathways of 2022 H7N3 HPAIVs in the new outbreak areas in Northern Mexico. We present genetic data of H7N3 viruses produced from 18 poultry farms infected in the spring of 2022. Our results indicate that the virus responsible for the current outbreak in Northern Mexico evolved from the Mexican lineage H7N3 HPAIV discovered in 2012. In the current outbreak, we identified five clusters of infection with four noticeably different genetic backgrounds. It is a cluster IV-like virus that was transmitted into one northern state causing an outbreak, then spreading to another neighboring northern state, possibly via a human-mediated mechanical transmission mechanism. The long-distance transmission event highlights the necessity for the more rigorous enforcement of biosafety measures in outbreaks. Additionally, we examined the evolutionary processes shaping the viral genetic and antigenic diversities. It is imperative to enhance active surveillance to include birds, the environment, and humans to detect HPAI in domestic poultry at an earlier point and eliminate it.

## 1. Introduction

Avian influenza viruses (AIVs) affect both domestic and wild birds, with sporadic cases being reported in several mammalian species, including humans [1]. Despite the identification of sixteen antigenically unique hemagglutinin (HA) subtypes of AIVs in birds, highly pathogenic forms of the virus have been restricted to H5 and H7 subtypes [2,3,4]. The process that leads to the conversion of H5 and H7 subtypes into highly pathogenic forms remains poorly understood. Highly pathogenic avian influenza (HPAI) viruses are molecularly characterized by the presence of multi-basic amino acids at the HA cleavage site or from nonhomologous recombination, which causes a foreign nucleotide sequence to be inserted into the cleavage site motif. HPAIV is a danger to poultry due to its tendency to cause severe systemic disease and mortality, resulting in heavy economic losses [5]. The consequences of HPAI on wild birds could potentially lead to a devastating effect on the biodiversity of ecosystems. In addition, HPAI can traverse the species barrier and infect mammals, including humans. The H7 subtype HA gene has been shown to appear in combination with all nine NA subtype genes. Poultry remains a significant source of human infection of HPAIV H7. The disease caused by H7 subtypes occurs worldwide, but different subtypes are more prevalent in certain regions than in others. There has been a transition in East Asia from H7N7 before 2013 to H7N9 thereafter. In Europe, H7N7 is the most common H7 subtype, whereas subtype H7N3 is mostly isolated from samples in North America [6]. These regionally specific dominant HA-NA subtypes are heavily associated with the evolution of subtypes within each reservoir [7,8].

Highly pathogenic H7N3 AIVs have remained in Mexican poultry since 2012, with two reported human infections in July 2012 [9]. Despite the implementation of vaccination, biosecurity, and surveillance measures, the identification of H7N3 HPAIV has expanded from its initial detection in the state of Jalisco in 2012 to an additional 15 states by 2021 (https://wahis.woah.org/#/dashboards/qd-dashboard, accessed on 7 September 2022). Historically, a total of 210 H7N3 HPAIV outbreaks were reported in Mexico since its first isolation in Jalisco in 2012 (https://wahis.woah.org/#/dashboards/qd-dashboard, accessed on 7 September 2022). Jalisco is the only state where H7N3 HPAIV has been detected on a yearly basis; it has had 98 outbreaks since 2012, with 3,408,353 cases reported to date. Guanajuato, a central state of Mexico, has experienced 46 H7N3 HPAI outbreaks, with 155,252 cases having been detected since 2013 (https://wahis.woah.org/#/dashboards/qd-dashboard, accessed on 7 September 2022). The H7N3 HPAI outbreak continues to expand to new areas. On 21 April 2022, the Mexico-United States Commission for the Prevention of Foot-and-Mouth Disease and other Exotic Animal Diseases (CPA) received a report of HPAIV cases in two farms from the General Cepeda municipality in Coahuila state. The investigation was conducted by members of the National Service for Health, Safety, and Agro-Alimentary Quality (SENASICA). Samples were collected and shipped to the SENASICA reference laboratory for diagnostic testing. Laboratory results indicated the presence of the H7 subtype AIV. On 26 April, positive H7 results were also obtained from the Gomez Palacios municipality in Durango state. The epidemiological tracing revealed a total of 78 production units that tested H7-positive, involving 74,463 susceptible birds and 543 deaths being reported. All affected farms were quarantined, and control measures were conducted, including depopulation, deep cleaning, disinfection, sanitary voids, and the use of sentinels. Including those birds during the last detection of H7 HPAIV on 30 June 2022, a total of 2.1 million birds from affected poultry production units and backyard farms have been culled. The states of Coahuila and Durango have recently been affected for the first time by H7 HPAI viruses. Full genome sequencing was conducted on selected isolates, revealing that the causative agent was H7N3 HPAIV.

Previous studies have indicated that Mexican H7N3 HPAIV originated from low-pathogenetic North American wild bird viral gene pools and subsequently mutated to a highly pathogenic phenotype by insertion of additional basic amino acids from host 28S ribosomal RNA (rRNA) in the HA cleavage site [10,11]. Since then, this virus has evolved rapidly and diverged into multiple clusters [12,13]. The outbreaks occur yearly, but no studies have been conducted on its transmission pathways in any previous outbreaks. Because of the rapid evolutionary dynamics of AIV, we hypothesize that sufficient genetic diversity may be produced during each outbreak to permit the reconstruction of the inter-farm transmission network, providing important insights into the implementation of efficient control measures.

In this study, we constructed a phylogenetic network of HPAIV H7N3 by using concatenated eight-gene segments of 10 full-genome sequences from new outbreak states (Coahuila and Durango) and 8 full-genome sequences from additional states reported to have H7N3 cases, taken from the Mexican surveillance system in 2022. To our knowledge, this is the first study that uses the phylogenetic approach to examine virus transmission pathways for HPAIV H7N3 between farms in Mexico. Furthermore, we studied the evolutionary processes, such as selection pressure and glycosylation, that might influence H7N3 genetic diversity and antigenic changes. Finally, we discuss the implications of our findings and offer recommendations to improve disease control.

## 2. Results

### 2.1. Phylogenetic Relationships

**Figure 1:**
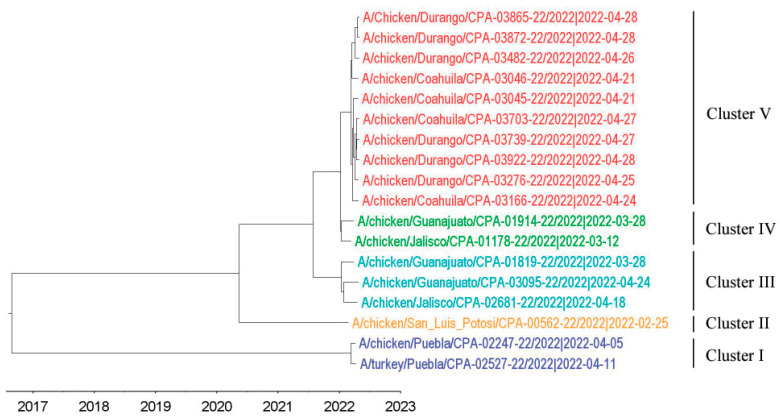
Maximum clade credibility tree inferred for the concatenated reading frames of the full genomes of 2022 Mexican H7N3 HPAIVs used in the current study. Sequences are color-coded as follows: blue, cluster I; yellow, cluster II; cyan, cluster III; green, cluster IV; red, cluster V.

Full-genome sequences of HPAI H7N3 viruses from the outbreak and surveillance systems (Table 1) were analyzed for their phylogenetic relationships. Using the maximum likelihood (ML) approach, the phylogenetic trees were constructed for the eight separate sequence datasets. The phylogenies from each dataset were grouped into the same five clusters (I to V) and showed the same topology for all eight segments (Figure S1 in the Supplementary Materials), indicating that no intra-subtype reassortment occurred. This allowed the concatenation of eight segments for phylogenetic transmission analysis by using a single alignment. The phylogenetic tree was also constructed from concatenated eight-gene segments, using a Bayesian Markov chain Monte Carlo (MCMC) method, as implemented in BEAST (Figure 1). The clusters formed were the same as those seen using ML approaches. Noticeably, cluster V contains solely those viruses from outbreak states. The time of the most recent common ancestor (TMRCA) for the entire dataset is estimated to be in early December of 2016, while clusters IV and V shared a TMRCA of mid-January, 2022. Viruses collected from outbreak states had a TMRCA of 15 March 2022. BLAST analyses did not show any close relationship between wild bird AIV gene segments and Mexican 2022 H7N3 AIV gene segments (data not shown). Details of the North American H7 HA (*n* = 680) and N3 NA (*n* = 585) genes were downloaded from IRD/GISAID (accessed on 22 August 2022). Analyses of these publicly available North American HA and NA sequences of H7N3 AIV with those of the Mexican H7N3 AIV in 2022 revealed that a single introduction of the virus from a wild bird virus to poultry in 2012 and all current viruses were the descendants of previous circulating viruses in Mexican domestic poultry (Figure S2 in the Supplementary Materials).

### 2.2. Phylogenetic Transmission Network

**Figure 2:**
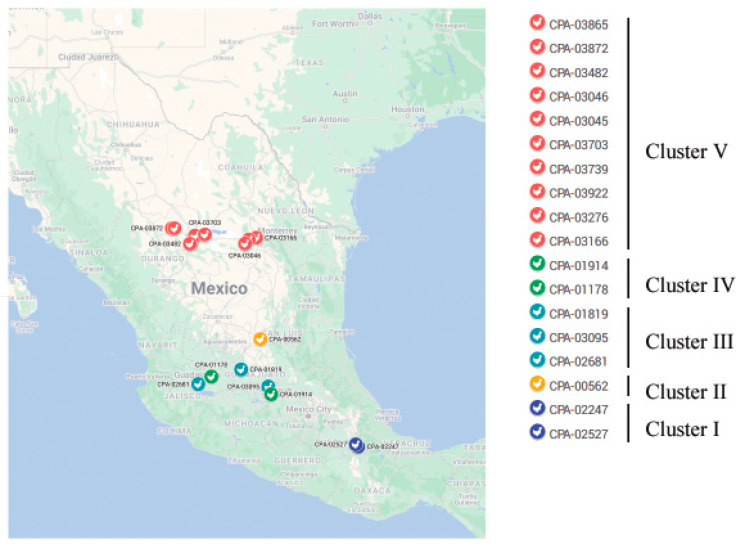
Map showing the location of different farms in Mexico where H7N3 cases were identified in 2022. The cases are color-coded in the same way as in Figure 1. The map was produced from Google Maps (Map data © 2022 Google; https://www.google.com/maps/place/Mexico (accessed on 27 July 2022), complying with the Terms of Service as outlined at http://www.google.ca/permissions/geoguidelines.html (accessed on 27 July 2022), with modifications.

**Figure 3:**
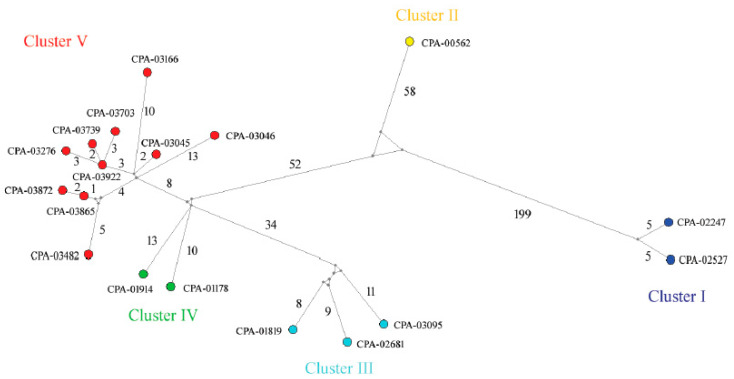
Median-joining phylogenetic network of the 2022 Mexican H7N3 HPAIVs. The median-joining network was constructed from the concatenated reading frames of 8 gene segments of each virus. This network includes all the most parsimonious trees linking the sequences. Each unique sequence is represented by a colored circle. The sequences are colored the same way as in Figure 1. Median vectors are shown in grey circles. The number of mutations is shown on the branch.

Figure 2 shows the spatial distribution of H7N3 viruses according to where they were detected. To assess the inter-farm and intra-farm transmission network, the amino acid sequences from concatenated eight-gene segments of each virus were used to construct a median-joining phylogenetic network (Figure 3). The network included all the parsimonious trees, allowing all the most plausible evolutionary pathways to link the farm samples. The network showed that the virus sequences were grouped into five clusters of infection, as is consistent with clusters identified with the gene-specific and concatenated phylogenetic trees. Sequences within the 5 clusters were separated, on average, by 23–69 nucleotide (nt) differences and 2–23 amino acid (aa) differences, whereas 78–1386 nt differences and 28–282 aa differences were observed between clusters. Two ancestral farm-sample sequences (CPA-03922 and CPA-03865) were observed in the network, thus representing two direct transmission events: (1) from CPA-03865 to CPA-03872; (2) CPA-03703, CPA-03739, and CPA-03276 were descended from CPA-03922.

### 2.3. Genetic Diversity in Mexican H7N3 HPAIVs

**Figure 4:**
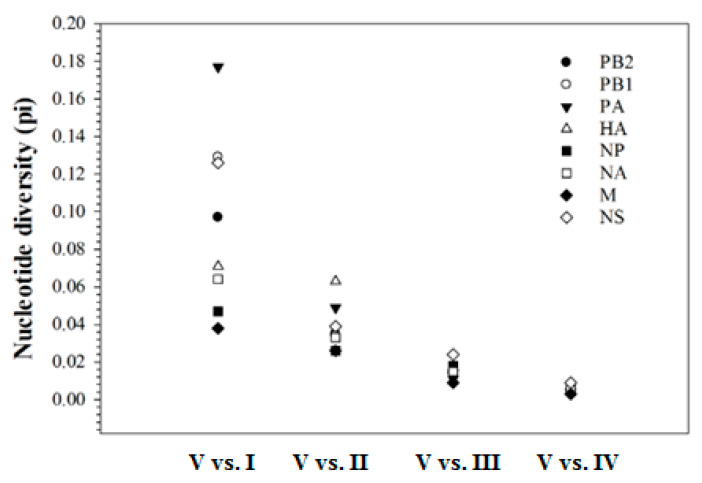
Nucleotide divergence between cluster V and clusters I–IV in the 2022 Mexican H7N3 HPAIVs. The diversity for eight segments decreases along with the increased number of clusters. The highest diversity is between cluster V and cluster I, whereas the lowest diversity is between cluster V and cluster IV. The calculation is based on the coding region for PB2, PB1, PA, HA, NP, and NA, while 982 nucleotides in the M segment and 838 nucleotides in the NS segment were used.

Analyses of the nucleotide distance between clusters revealed that a high level of genetic diversity was observed between cluster I viruses and cluster II–V viruses, ranging from 9.4% to 17.7% on PB2, PB1, PA, and NS segments, and 3.4% to 7.7% on HA, NP, NA, and M segments. This is followed by a distance of 2.2% to 6.5% between cluster II viruses and clusters III–V viruses, and a distance of 1.1% to 2.4% between cluster III viruses and clusters IV–V viruses. The least genetic divergence was observed between clusters IV and V, ranging from 0.3% to 0.9%. Figure 4 shows an example of divergence between cluster V and clusters I–IV. Genetic relatedness increased with each successive cluster.

Examining the H7N3 virus genomes revealed 36 molecular markers/motifs that were previously associated with the phenotypic change of AIVs (Table 2). Twenty-five (69%) of the markers/motifs were present in the H7N3 AIV genome (i.e., they were present in all the H7N3 AIV genomes examined). The majority of these markers were associated with either increased polymerase activity in avian and mammalian cell lines and/or virulence in the experimental animal models.

### 2.4. Selection Pressures Acting on the Viral Genes

Our analysis reveals that the HA, NA, NS1, and NS2 genes have more nonsynonymous substitutions per site than those in PB2, PB1, PA, NP, M1, and M2 genes (Figure S3 in the Supplementary Materials). Mean ratios of nonsynonymous to synonymous evolutionary changes (dN/dS) for the major coding regions in 2022 H7N3 HPAIVs ranged from 0.06 to 0.35, indicating purifying selection. When using SLAC (single-likelihood ancestor counting), FEL(fixed-effects likelihood), and MEME (mixed-effects model of evolution) methods (see Materials and Methods), we did not identify any positively selected residues. However, when using FUBAR (fast, unconstrained Bayesian approximation), we found five positively selected residues with a posterior probability of >0.9 (i.e., HA-151, HA-343, NA-58, M1-36, and NS1-209). Residue 151 in the HA gene is one of the amino acids that constitutes antigenic site A, whereas residue 343 in the HA gene is located within the cleavage site motif. This could result in the mutation of a non-basic amino acid to a basic amino acid or vice visa. The biological function of residue 58 in the NA gene is unknown, but residue 36 in the M1 gene is located in the N-terminal region that binds to the viral ribonucleoprotein complex (vRNP). Residue 209 in the NS1 gene is located in the unstructured flexible tail, which contains several motifs, including CDK/ERK phosphorylation, Crk/CrkL SH3 binding, the PDZ ligand, and NoLS/NLS2.

### 2.5. Antigenic Evolution of HA Gene

**Figure 5:**
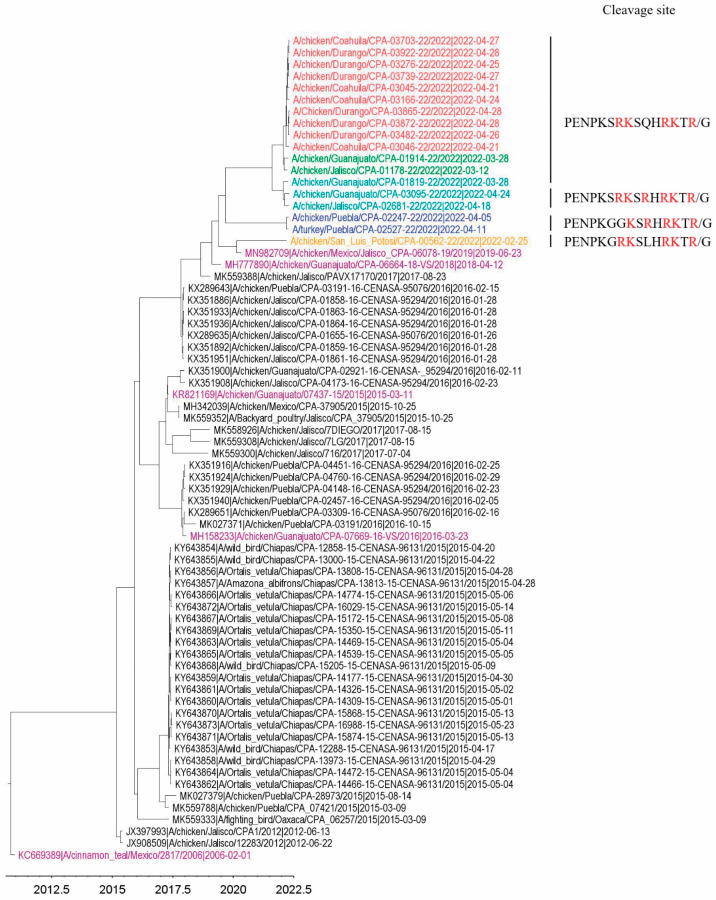
Maximum clade credibility tree, inferred for the H7 HA gene from the available Mexican H7N3 AIVs. Sequences in 2022 are color-coded in the same way as in Figure 1. The five historical vaccine strains are shown in purple. The cleavage site motif is only shown for the 2022 H7 HA sequences. Multiple basic amino acids are highlighted in red at the cleavage site.

We compared the genetic distances of 18 H7 HA nucleotide sequences from 2022 with those that are publicly available in the GenBank/GISAID database in Mexico (*n* = 52, accessed on 22 August 2022) and found out that the genetic distance between the HA sequences of 2022 and 2012–2017 was 8%, whereas 4% was observed with those from 2018–2019. Figure 5 shows that the 2022 Mexican H7 HA evolved from recently circulating viruses to multiple clusters. In the dataset of H7 HA (*n* = 70), all HA genes contained a highly pathogenic character, with 5–7 critical basic amino acid sequences at the cleavage site motif, except forA/cinnamon teal/Mexico/2817/2006, which contained only one critical basic amino acid at the cleavage site (low pathogenic phenotype).

**Figure 6:**
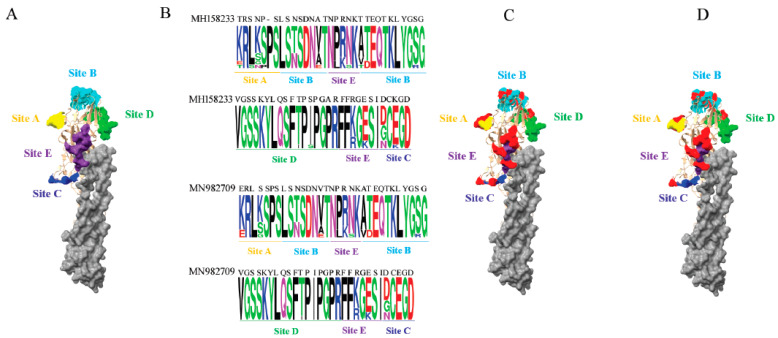
Antigenic variations identified in the H7 HA proteins in the 2022 Mexican H7N3 HPAIV. (**A**) The known antigenic sites are color-coded and are shown in the globular head of H7 HA. (**B**) SeqLogo analysis of amino acid substitutions at known antigenic sites of the HA protein against HA proteins of the inactivated vaccine strain, A/chicken/Guanajuato/CPA-07669-16-VS/2016 (accession no. MH158233), recombinant vaccine strain, A/chicken/Mexico/Jalisco-CPA-06078-19/2019 (accession no. MN982709). The sequence logo consists of stacks of symbols for the corresponding amino acids at antigenic sites. (**C**) Substituted antigenic residues are shown in red, compared with antigenic residues of the inactivated vaccine strain. (**D**) Substituted antigenic residues are shown in red, compared with antigenic residues of the recombinant vaccine strain. The H7 HA crystal structure is manipulated with Chimera and is shown in the surface format, using the structure from A/turkey/Italy/214845/2002 (PDB # 4BSG). Antigenic sites are denoted as follows: yellow, site A; cyan, site B; blue, site C; green, site D; purple, site E.

Since the vaccination program was implemented in 2012, the Mexican authorities have updated the H7 vaccines five times, according to the records. After analyzing the five known antigenic sites on the 2022 H7N3 HA proteins against five H7 vaccines used in Mexico, we found amino acid changes corresponding to the phylogenetic distance observed in the H7 phylogenetic tree (i.e., the closer the phylogenetic distance to the 2022 H7 HA genes, the fewer the antigenic alterations (Figure 5 and Figure S4 in the Supplementary Materials)). For example, the HA antigenic sites of the inactivated vaccine strain, A/chicken/Guanajuato/CPA-07669-16-VS/2016, and those of the recombinant vector vaccine, A/chicken/Guanajuato/CPA-06078-19/2019, were analyzed with the HA antigenic sites of 2022 H7N3 viruses using the SeqLogo analysis tool. The locations of the amino acid substitutions are shown in the three-dimensional structure of the H7 HA protein (Figure 6).

We also analyzed the glycosylation in the 2022 H7 HA proteins since glycosylation is a strategy commonly used by influenza viruses to mask their antigenic sites from being recognized by the immune system. Our analysis shows that four glycosylation sites were fixed in all 2022 H7 HA proteins with sequons of NATE, NGTS, NDTV(I), and NNTY. Two glycosylations (NYSG and NLSY) were not found in any official H7 vaccines despite their low abundance. Two glycosylations (NMTL(R) and NLSY) located in the receptor binding domain are near the antigenic sites B and E (Table 3).

## 3. Discussion

Our findings from 18 H7N3 AIV genomes sampled in Mexico in the spring of 2022 demonstrate the value of using sequencing for outbreak investigation and surveillance. Our analysis reveals that all 2022 H7N3 viruses responsible for the HPAI outbreaks belonged solely to the Mexican lineage H7N3 viruses, demonstrating that these outbreaks are a reoccurring event. These viruses have genetically drifted further from the USA and Canadian H7N3 viruses. The results obtained in this study demonstrate that substantial viral genetic diversity can be acquired within a brief time span during an HPAI outbreak, allowing the use of genome sequences to determine virus transmission dynamics with phylogenetic analyses.

The 2022 Mexican H7N3 viruses were phylogenetically grouped into 5 clusters without evidence of reassortment. This allowed us to explicitly concatenate all eight gene segments for transmission network analysis. Phylogenetic analyses revealed five clusters of infections with four noticeable different genetic backgrounds: (1) source #1 occurred in San Luis Potosi in February; (2) source #2 occurred in March and involved Jalisco and Guanajuato, from where the viruses were transmitted to Coahuila and Durango; (3) source #3 occurred in Puebla in early April; (4) source #4 occurred in Jalisco and Guanajuato in late April. The viruses within the same source of infection contained the same cleavage site motif in the HA protein, whereas differences were observed between infections. We did not observe wild bird-associated introductions; rather, they are descendants of the ancestral H7N3 HPAIV that emerged in 2012, according to the HA and NA ML trees (Figure S2 in the Supplementary Materials). Richard et al. showed that the emergence of HPAIV H7 seems to be primarily related to the adaptation of LPAIV to poultry [57]. There lacks proof that H7 HPAIVs have ever emerged in wild birds [58]. It remains poorly understood that H7N3 HPAIV has persisted in Mexico for a decade despite surveillance, vaccination, and biosecurity measures being in place.

In this study, huge genetic distances were observed between cluster I and the other 4 clusters (~11%), followed by distances between cluster II and clusters III–IV(~4%), then between cluster III and clusters IV–V (~2%); the mean nucleotide identity between clusters IV and V was high (99.5%). Given the fact that the viruses Jalisco/CPA—01178 and Guanajuato/CPA-01914 shared the same ancestral virus indicated by M-J network analysis and the timing of infection from the epidemiological data (6 days apart), it was hypothesized that Guanajuato/CPA-01914 was derived from Jalisco/CPA-01178, possibly through the movement of poultry or human-mediated mechanical transport. Possibly by the same mechanism, cluster IV-like viruses were transmitted to Coahuila and spread to Durango. The viruses collected from these two states showed 13 amino acid differences on average in the entire genome, thus forming cluster V. This quick spread of viruses in Coahuila and Durango is most likely associated with the highly naïve poultry populations since no H7 vaccination has been administrated previously. Two direct transmission events identified in the M-J network (CPA-03865 to CPA-03872; CPA-03922 to CPA-03703, CPA-03276, and CPA-03739) are also possibly associated with poultry movements and other human-mediated mechanisms, but airborne spread cannot be ruled out because of their closer geographic proximity. Human-mediated virus transmissions are commonly reported [59,60]. Examples of such human-mediated transmissions are associated with but are not limited to the sharing of equipment and poultry crews and the unauthorized movement of birds or their products, which could be avoided by better enforcement and more widely distributed biosafety instructions and training.

TMRCA estimations suggest that the H7N3 virus may have been presented in poultry weeks prior to the first mortality being reported in Coahuila. This is consistent with the epidemiological models based on mortality data, showing that approximately two weeks can elapse after the introduction of HPAIV in a flock before the change in mortality is observed [61]. This also suggests that the HPAI H7N3 virus was very stable and well-adapted to poultry when the outbreak started. Furthermore, examining the coding region of the 2022 HPAI H7N3 genome revealed that many amino acid changes associated with increased polymerase activity and replication in mammalian cells appeared already fixed in the population during the outbreak. These results imply that the early and regular monitoring of poultry farms necessitates the detection and containment of avian influenza viruses prior to a potential risk to animal and public health. Indeed, two confirmed cases of human infection of H7N3 HPAIV were reported from poultry workers in July 2012 when the first outbreak of HPAI H7N3 occurred in the state of Jalisco, Mexico, in June 2012 [9].

We did not observe identical sequences in the network, indicating that viruses underwent rapid evolution. However, analyses of the selection pressure showed that virus evolution was mainly driven by purifying selection pressure, with only a limited amount of site-specific positive selection pressure points identified in the HA, NA, M1, and NS1 genes. Notably, one positively selected residue is located within antigenic site A of the HA protein; this would lead to antigenic change, hindering the effectiveness of vaccination.

The antigenic evolution of the HA protein in 2022 Mexican H7N3 HPAIV was assessed by comparison of the alteration of known antigenic residues with that of recent inactivated (A/chicken/Guanajuato/CPA-07669-16/2016) and recombinant vector (A/chicken/Guanajuato/CPA-06078-19/2019) H7 vaccines. A total of 18 amino acid alterations were observed when compared with the inactivated vaccine, whereas 14 amino acid changes were found when compared with the recombinant vector vaccine. This suggests that the current vaccines need to be reevaluated for their protective efficacy against the 2022 HPAI H7N3 strains. Analysis of glycosylation in the HA protein of 2022 Mexican H7N3 viruses revealed that 6 out of 9 glycosylated residues are located in the receptor binding domain, while two glycosylated residues in the receptor binding domain were not found in any H7 vaccines. Notably, the newly glycosylated residues are close to antigenic sites B and E. Immune pressure has played a vital role in viral antigenic evolution and residue glycosylation. The same scenario has been shown in the Mexican H5N2 LPAIV evolutionary analysis [62].

In summary, viral genetic data, complemented with epidemiological studies, allow the identification of clusters of infections and specific farm-to-farm transmission events. A combination of evolutionary processes, such as mutations caused by immune pressure, and positive selection has driven the genetic diversity observed in the 2022 HPAIV H7N3 outbreak in Mexico.

To ultimately eradicate HPAI in domestic poultry, enhanced active surveillance, education, biosecurity, and rapid molecular diagnostics will facilitate early detection and elimination.

## 4. Materials and Methods

### 4.1. Virus Isolation and Genome Sequencing

A total of 18 samples sequenced in this study were derived from H7N3 AIVs that were isolated from Mexican poultry in 2022 (Table 1). These samples were processed at the animal health diagnostic laboratories of the Animal Health General Directorate, Animal and Plant Health, Food Inspection, and Food Safety National Services (SENASICA), Mexico. All the procedures for AIV isolation in chicken embryonated eggs were approved by the Care and Use of Animals Committee of the SENASICA, based on the guidelines established by the Official Mexican Standard NOM-062-ZOO-1999 and the Internal Committee for the Care and Use of Laboratory Animals (CICUAL) belonging to the CPA-SENASICA (authorization number CICUAL-CPA-002-2022). Viral RNA extraction was performed using the High Pure RNA isolation kit (Roche Diagnostics, Laval, QC, Canada) according to the manufacturer’s protocol. The entire genome (PB2, PB1, PA, HA, NP, NA, M, and NS) of each virus was amplified as described previously [63]. Illumina MiSeq Technology was used for full-genome sequencing. The Nextera XT DNA Sample Preparation Kit (Illumina, San Diego, CA, USA) was used to generate multiplexed paired-end sequencing libraries, according to the manufacturer’s instructions. Each genome segment was assembled utilizing the DNAstar SeqMan NGen software (Version 17; DNASTAR, Inc., Madison, WI, USA).

### 4.2. Nucleotide Sequences Used in the Study

All publicly available complete sequences of HA and NA of North American H7N3 AIVs were downloaded from the Influenza Research Database (IRD, https://www.fludb.org/, accessed on 22 August 2022) and GISAID (https://gisaid.org, accessed on 22 August 2022). This led to the datasets of 585 NA and 680 HA when combined with sequences generated in this study.

### 4.3. Sequence Analyses

The nucleotides in the coding regions of each segment were aligned using the Muscle program in MEGA-X [64]. To infer phylogenetic trees of North American HA and NA, the maximum likelihood (ML) approach was applied using RAxML v8.2.12 [65], with a GTR+G substitution model and 1000 bootstrap replicates. Trees were rooted at the midpoint. ML trees of eight segments of 18 Mexican H7N3 AIVs were generated in MEGA-X [64].

A Bayesian approach was implemented to explore additional evolutionary information. The times to the most recent common ancestor (tMRCA) of the 2022 Mexican H7N3 AIV strains were estimated for concatenated gene segments using the Bayesian Markov chain Monte Carlo (BMCMC) method in the program BEAST, version 2.6.7 [66]. The best-fit nucleotide substitution model was determined for each gene segment by MEGA-X software (Philadelphia, PA, USA) [64]. An uncorrelated log-normal relaxed molecular clock model was used for the HA of 70 Mexican H7N3 AIVs. The age of the viruses was defined as the date of sample collection. For the dataset, at least two independent BEAST analyses were run for 50 million generations, sampling every 5000 steps. Convergences and effective sample sizes (ESS) of the estimates were checked using Tracer v1.7.2 (http://tree.bio.ed.ac.uk/software/tracer (accessed on 25 July 2022)) to ensure ESS values of >200.

### 4.4. Transmission Network Construction

The concatenated eight-gene segments that encode the eleven proteins of 2022 Mexican HPAIV H7N3 (PB2, PB1, PB1-F2, PA, PA-X, HA, NA, M1, M2, NS1, and NS2) were used to construct a phylogenetic network, using the median-joining method implemented in the program Network v10.2.0.0 (http://www.fluxus-engineering.com (accessed on 25 July 2022)). The parameter epsilon, which controls the level of homoplasy, was set at the same value as the weight of characters used to calculate the genetic distances (weight value = 10).

### 4.5. Analysis of Selection Pressure

Site-specific selection pressures for all segments of the Mexican H7N3AIVs were measured as nonsynonymous (dN)–synonymous (dS) nucleotide substitutions per site. The differences were estimated using the SLAC (single-likelihood ancestor counting) [67], FEL (fixed-effects likelihood) [67], MEME (mixed-effects model of evolution) [68], and FUBAR (fast, unconstrained Bayesian approximation) [69] methods available at the Datamonkey website [70]. A cut-off *p*-value to classify a site as positively or negatively selected was set at 0.1 for SLAC and 0.01 for FEL and the MEME methods. The cut-off value for the posterior probability in the FUBAR method was set at 0.9, to reflect a positive or negative selection at a given site.

### 4.6. 3D Structural Analyses

Molecular graphics coordinates of the H7 HA crystal structure (PDB #4BSG [71]) from A/turkey/Italy/214845/2002 were performed using the UCSF Chimera package from the Resource for Biocomputing, Visualization, and Informatics at the University of California, San Francisco [72]. The resulting images were imported into Adobe Photoshop for editing.

### 4.7. Antigenic and Glycosylation Analyses of HA Protein

The amino acids of the 2022 Mexican H7 HA protein at the antigenic sites were compared with those of the inactivated vaccine strain, A/chicken/Guanajuato/CPA-07669-16-VS/2016 (accession no. MH158233) or recombinant vaccine strain, A/chicken/Guanajuato/CPA-06078-19/2019 (accession no. MN982709). The variations were visualized by the SeqLogo, implemented in TBtools [73]. The sequence logo consists of stacks of symbols for the corresponding amino acids. The potential N-glycosylation sites of the H7 HA protein were predicted using the NetNGlyc 1.0 server (http://www.cbs.dtu.dk/services/NetNGlyc (accessed on 20 August 2022)).

### 4.8. Nucleotide Sequence Accession Numbers

All sequence data generated in this study have been submitted to GenBank and assigned the accession numbers OP535168 to OP535311.

## Figures and Tables

**Table 1:** Mexican H7N3 AIVs, collected in 2022 and used in the study.

Strain	Collection Date (y/m/d)	Type of Surveillance	Type of Sample Bird	Farm ID	Network ID	HA Cleavage Site Motif
A/chicken/San Luis Potosí/CPA-00562-22/2022	2022-02-25	Passive	Sick	Farm A	CPA-00562	PENPKGRKSLHRKTR/G
A/chicken/Jalisco/CPA-01178-22/2022	2022-03-12	Passive	Dead corpse	Farm B	CPA-01178	PENPKSRKSQHRKTR/G
A/chicken/Coahuila/CPA-03045-22/2022	2022-04-21	Passive	Dead corpse	Farm H	CPA-03045	PENPKSRKSQHRKTR/G
A/chicken/Coahuila/CPA-03046-22/2022	2022-04-21	Passive	Dead corpse	Farm I	CPA-03046	PENPKSRKSQHRKTR/G
A/chicken/Puebla/CPA-02247-22/2022	2022-04-05	Passive	Dead corpse	Farm E	CPA-02247	PENPKGGKSRHRKTR/G
A/turkey/Puebla/CPA-02527-22/2022	2022-04-11	Passive	Dead corpse	Farm F	CPA-02527	PENPKGGKSRHRKTR/G
A/chicken/Guanajuato/CPA-01819-22/2022	2022-03-28	Active	Healthy	Farm C	CPA-01819	PENPKSRKSRHRKTR/G
A/chicken/Guanajuato/CPA-01914-22/2022	2022-03-28	Active	Healthy	Farm D	CPA-01914	PENPKSRKSQHRKTR/G
A/chicken/Jalisco/CPA-02681-22/2022	2022-04-18	Passive	Sick	Farm G	CPA-02681	PENPKSRKSRHRKTR/G
A/chicken/Guanajuato/CPA-03095-22/2022	2022-04-24	Passive	Sick	Farm J	CPA-03095	PENPKSRKSRHRKTR/G
A/chicken/Coahuila/CPA-03166-22/2022	2022-04-24	Active	Dead corpse	Farm K	CPA-03166	PENPKSRKSQHRKTR/G
A/chicken/Durango/CPA-03482-22/2022	2022-04-26	Active	Dead corpse	Farm M	CPA-03482	PENPKSRKSQHRKTR/G
A/Chicken/Durango/CPA-03865-22/2022	2022-04-28	Active	Healthy	Farm P	CPA-03865	PENPKSRKSQHRKTR/G
A/chicken/Coahuila/CPA-03703-22/2022	2022-04-27	Active	Healthy	Farm N	CPA-03703	PENPKSRKSQHRKTR/G
A/chicken/Durango/CPA-03739-22/2022	2022-04-27	Active	Dead corpse	Farm O	CPA-03739	PENPKSRKSQHRKTR/G
A/chicken/Durango/CPA-03872-22/2022	2022-04-28	Active	Dead corpse	Farm Q	CPA-03872	PENPKSRKSQHRKTR/G
A/chicken/Durango/CPA-03922-22/2022	2022-04-28	Active	Dead corpse	Farm R	CPA-03922	PENPKSRKSQHRKTR/G
A/chicken/Durango/CPA-03276-22/2022	2022-04-25	Active	Sick	Farm L	CPA-03276	PENPKSRKSQHRKTR/G

**Table 2:** Molecular markers/motifs in AIVs previously associated with phenotypic changes.

Gene	Position ^a^/Residue/Motif (%) ^b^	Phenotype [Reference Number]
PB2	292V (89)	Increased polymerase activity in mammalian cell line and increased virulence in mice [14]
	389R (100)	Increased polymerase activity and replication in mammalian cell line [15]
	526R (6)	Increased polymerase activity in mammalian cell line [16]
	598T (100)	Increased polymerase activity and replication in mammalian cells and increased virulence in mice [15]
	627E (100)	Increased virulence in chickens [17]
	715N (89)	Decreased virulence in mice [18]
	89V, 309D (11)	Increased polymerase activity in mammalian cell line and increased virulence in mice [19]
PB1	3V (100)	Increased polymerase activity and viral replication in avian and mammalian cell lines [20]
	622G (100)	Increased polymerase activity and virulence in mice [21]
	678N (6)	Increased replication in avian and mammalian cell lines [22]
PB1-F2	66S (100)	Enhanced replication, virulence and antiviral response in mice [23,24]
PA	37A (100)	Increased polymerase activity in mammalian cell line [25]
	63I (89)	Increased polymerase activity and enhanced replication in mammalian cell line, increased virulence in mice [26,27]
	190S (100)	Decreased virulence in mice [28]
	383D (100)	Increased polymerase activity in mammalian and avian cell lines [29,30]
	400P (89)	Decreased virulence in mice [28]
	409S (100)	Increased polymerase activity and replication in mammalian cell line [25]
	497R (6)	Increased polymerase activity in mammalian cell line [31]
HA	126N (6)	Increased virus binding to α2-6 [32]
	214I (6)	Increased virus binding to α2-6 [33]
	326 to 329 (100)	Polybasic cleavage motif sequence required for high pathogenicity avian influenza viruses [34,35,36]
	393E (100)	Increased pH of fusion, decreased HA stability, decreased virulence in mice [37]
NP	184K (100)	Increased replication in avian cells and virulence in chickens enhanced IFN response [38]
NA	117T (100)	Reduced susceptibility to oseltamivir and zanamivir [39]
M1	30D (100)	Increased virulence in mice [40]
	43M (100)	Increased virulence in mice, chickens, and ducks [41]
	215A (100)	Increased virulence in mice [40]
M2	31N (100)	Increased resistance to amantadine and rimantadine [42,43,44,45,46,47,48]
NS1	42S (100)	Increased virulence and decreased antiviral response in mice [49]
	106M (100)	Increased viral replication in mammalian cells and increased virulence in mice [50]
	138F (100)	Increased replication in mammalian cells, decreased interferon response [51]
	149A (100)	Increased virulence and decreased interferon response in chicken [52]
	103F, 106M (100)	Increased virulence in mice [53,54]
	3S, 41K (11)	Enhanced replication in mammalian cells and pathogenicity in mice [55]
	55E, 66E, 138F (100)	Enhanced replication in mammalian cells, decreased IF response [51]
	ESEV (227-230, PDZ domain) (100)	Increased virulence in mice Decreased viral replication in mammalian and avian cell lines Increased viral replication and virulence in mice decreased viral replication in human and duck cell lines [56]

^a^ Codon position. H3 numbering is used for HA; N2 numbering is used for NA; internal genes are numbered according to alignment with A/Goose/Guandong/1/1996 (H5N1). ^b^ The percentage of each amino acid in the dataset is shown in parentheses.

**Table 3:** Potential N-glycosylation sites identified in the HA protein of the 2022 Mexican H7N3 HPAIVs.

Amino Acid Position ^a^	Structural Location	Sequons ^b^	% of Isolates
30 (22)	stalk	NGTK	44
46 (38)	stalk	NATE	100
134 (126)	RBD	NYSG *	6
141 (133)	RBD	NGTS	100
166/167 (n/a)	RBD	NVTF	83
171/172/173 (162/163/164)	RBD	NMTL(R)	83
174 (165)	RBD	NLSY *	17
248/249/250 (239/240/241)	RBD	NDTV(I)	100
500/501/502 (482/483/484)	stalk	NNTY	100

^a^ H3 numbering is in parentheses. n/a, not applicable. ^b^ The glycosylated residue is shown in red. *, not found in vaccine HA proteins. RBD, receptor binding domain.

## Data Availability

Eighteen full-genome sequences were obtained from active/passive surveillance in Mexican poultry, which have GenBank accession numbers OP535168-OP535311.

## Supplementary Materials

The following supporting information can be downloaded at: https://www.mdpi.com/article/10.3390/pathogens11111284/s1, Figure S1: ML tree of the individual gene segment of 2022 Mexican H7N3 HPAIV. Figure S2: ML trees inferred from available North American H7N3 AIVs. Figure S3: Mean dN/dS values for major coding regions of 2022 Mexican H7N3 HPAIVs. Figure S4: The number of amino acid alterations at known antigenic sites of H7 HA protein in 2022 Mexican H7N3 HPAIVs.
