# Supplementary material for: Phylogenetic Inference of the 2022 Highly Pathogenic H7N3 Avian Influenza Outbreak in Northern Mexico
Source: Pathogens. 2022 Nov 1;11(11):1284. doi: 10.3390/pathogens11111284 (PMC9692817; doi:10.3390/pathogens11111284)
Supplement: Supplementary file 1 [file pathogens-11-01284-s001.zip › pathogens-1971936-supplementary.pdf]

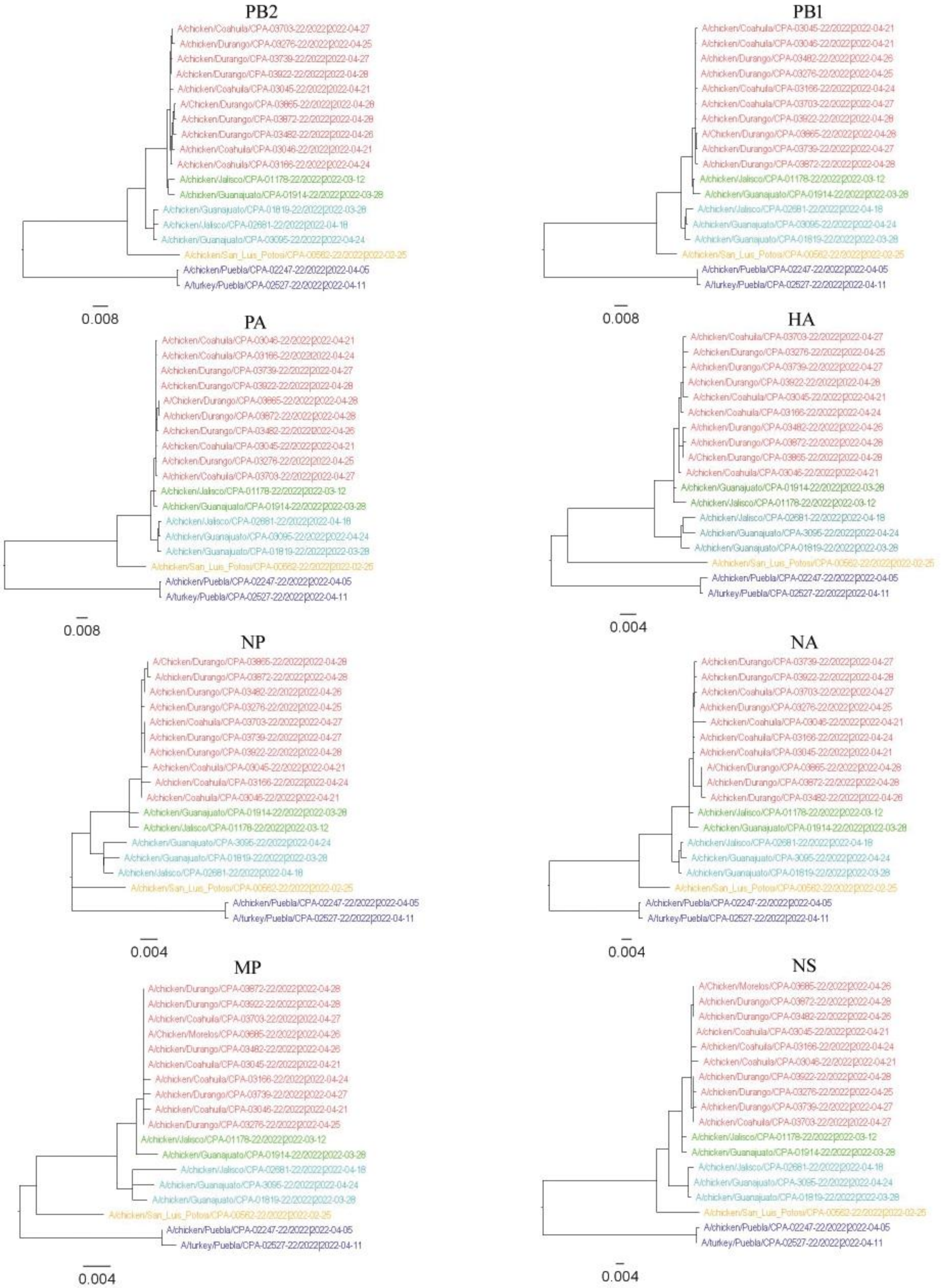

Figure S1. ML tree of the individual gene segment of 2022 Mexican H7N3 HPAIV. Clusters are color-coded as follows: blue, cluster Ⅰ; yellow, cluster Ⅱ; cyan, cluster Ⅲ; green, cluster Ⅳ; red, cluster Ⅴ.

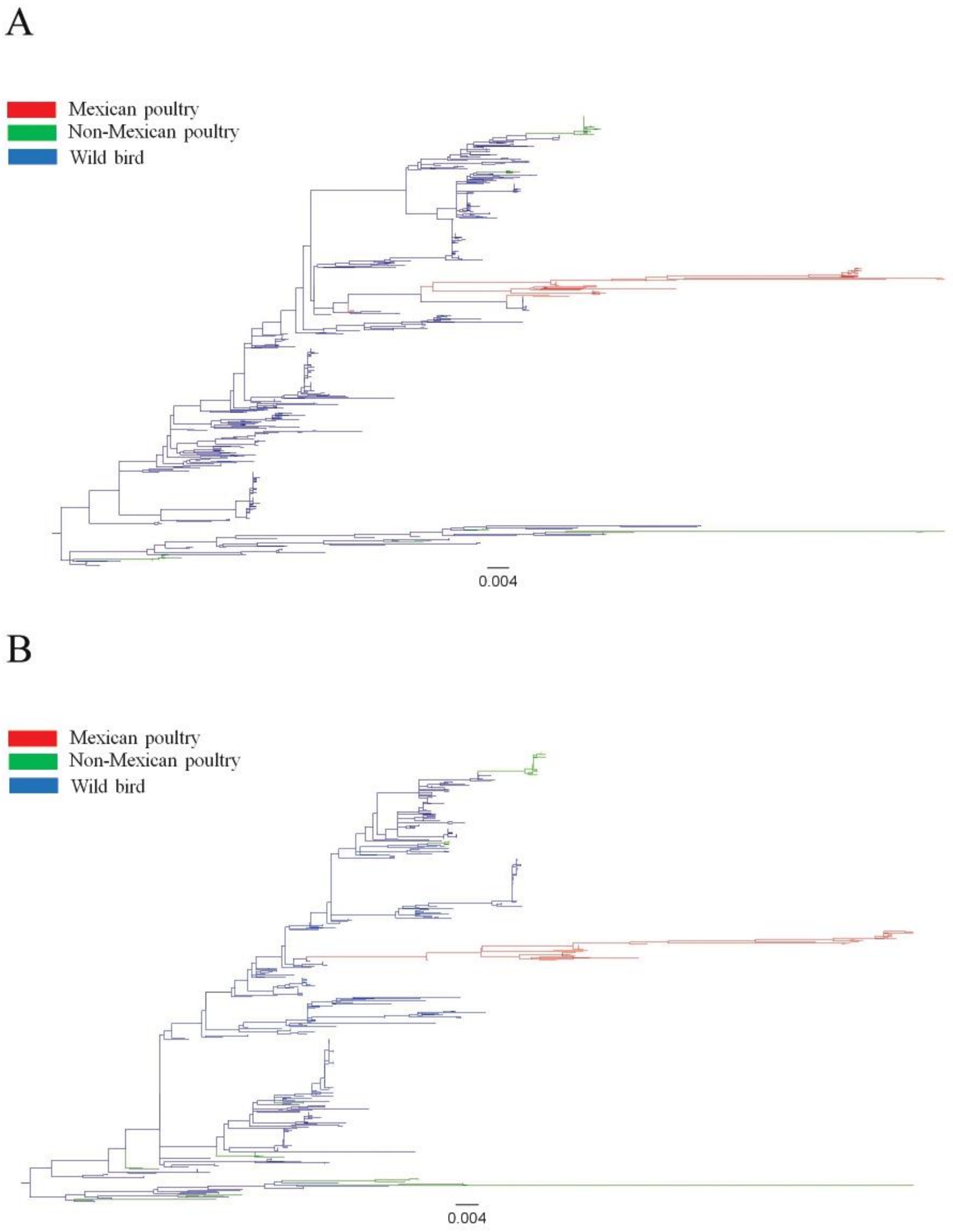

Figure S2. ML trees inferred from available North American H7N3 AIVs. (A) HA phylogenies for the complete coding region from 680 H7N3 AIVs. (B) NA phylogenies for the complete coding region from 583 H7N3 AIVs. Sources of the virus are denoted as follows: red Mexican poultry; green, non-Mexican poultry; blue, wild bird.

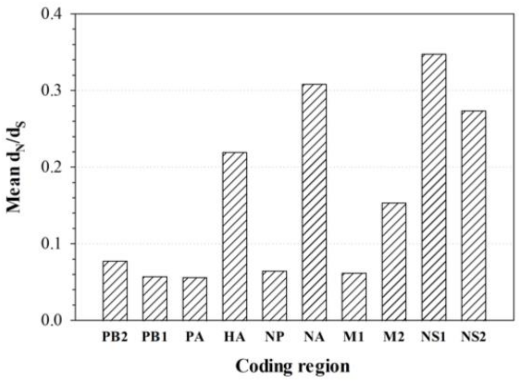

Figure S3. Mean dN/ds values for major coding regions of 2022 Mexican H7N3 HPAIVs.

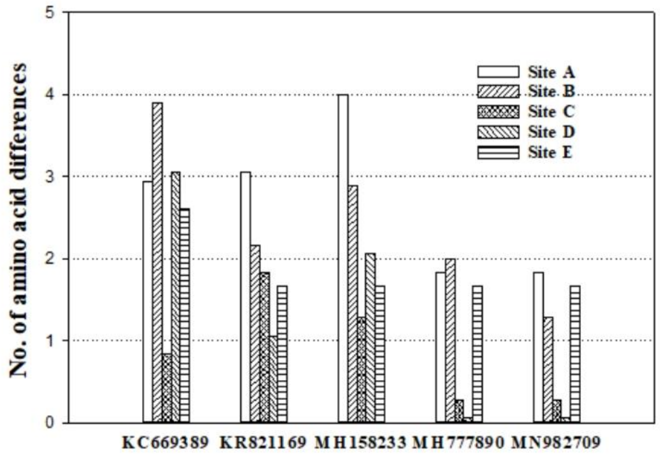

Figure S4. The number of amino acid alterations at known antigenic sites of H7 HA protein in 2022 Mexican H7N3 HPAIVs. The substituted residues were determined against each of the historical vaccine HA proteins. The denotations for vaccine strain are as follows: KC669389, A/cinnamon teal/ Mexicoo/2817/2006; KR821169, A/chicken/Guanajuato/07437-15/2015; MH158233, A/chicken/Guanajuato/CPA-07669-16-VS/2016; MH777890, A/chicken/Guanajuato/CPA-06664-18-VS/2018; MN982709, A/chicken/Mexico/Jalisco-CPA-06078-19/2019.
